# Time from trophectoderm biopsy to vitrification affects the developmental competence of biopsied blastocysts

**DOI:** 10.1002/rmb2.12439

**Published:** 2022-01-29

**Authors:** Tetsuya Miki, Kenji Ezoe, Shizu Kouraba, Kazuki Ohata, Keiichi Kato

**Affiliations:** ^1^ Kato Ladies Clinic Tokyo Japan; ^2^ Towako Medical Research Center Ishikawa Japan

**Keywords:** blastocyst, outgrowth, re‐expansion, trophectoderm biopsy, vitrification

## Abstract

**Purpose:**

The present study aimed to examine the correlations of the time interval from trophectoderm (TE) biopsy to vitrification with the blastocyst survival rate and blastocyst outgrowth ability.

**Methods:**

A total of 1,202 mouse blastocysts were randomly divided into control (non‐biopsy) and TE biopsy groups. The biopsied blastocysts were vitrified at various time points. The survival rate after warming, blastocyst adhesion rate, and outgrowth area was investigated. Several biopsied blastocysts were cultured in a time‐lapse incubator, and the time required for re‐expansion was measured.

**Results:**

Blastocyst survival rates after warming and blastocyst adhesion rates were comparable between the control and biopsy groups. The area of trophoblast outgrowth in the 1‐h biopsy group was significantly smaller than that in the control, 0‐h biopsy, and 4‐h biopsy groups (*p* = 0.0304, *p* = 0.0058, and *p* = 0.0029, respectively). Re‐expansion of blastocysts was observed at a high incidence 1–2 h after TE biopsy.

**Conclusions:**

The vitrification of biopsied blastocysts in the process of re‐expansion impairs outgrowth competence; therefore, blastocyst vitrification should be performed immediately after TE biopsy and before initiation of re‐expansion.

## 1 INTRODUCTION

Preimplantation genetic testing (PGT) is primarily conducted among patients undergoing in vitro fertilization (IVF) to increase pregnancy rates per embryo transfer, decrease the miscarriage rate, and reduce time to pregnancy.^1^, ^2^ Embryonic biopsies are performed at the cleavage or blastocyst stage according to each institutional policy, although blastocyst or trophectoderm (TE) biopsy is currently the most widely used technique.^3^, ^4^ After opening the zona pellucida using a laser system, some TE cells—which play a crucial role in implantation^5^—are removed from the blastocyst. The blastocysts are then cryopreserved until the genetic testing results are available. In contrast, there is a concern about whether TE biopsy adversely affects blastocyst viability and subsequent attachment competence to the uterus. Previous studies have reported that various factors, including the biopsy procedure, affect the survival rate after vitrification^6^, ^7^, ^8^; therefore, optimization of the biopsy protocol is required in each institution.

In the present study, we focused on the effect of time interval from TE biopsy to vitrification on blastocyst viability after warming. Several studies have previously assessed the effect of these time intervals. One such study reported that blastocyst vitrification should be performed 3 or more hours after TE biopsy.^9^ However, other groups have reported that the blastocysts should be vitrified immediately or within 1 h after TE biopsy^1^, ^10^; therefore, the optimal timing of vitrification after TE biopsy remains controversial. In the present study, we examined the correlation of different time intervals from TE biopsy to vitrification with blastocyst survival rate and blastocyst outgrowth ability using a mouse model.

## 2 MATERIALS AND METHODS

### 2.1 Animals

All mice were housed in an isolator for experimental animals (SLC Inc.) under a 12‐h light/dark cycle, as previously reported.^11^ All mouse experiments were conducted in accordance with the guidelines of the Institutional Animal Care and Use Committee of Kato Ladies Clinic, which are based on the Animal Welfare Act Regulations and the Guide for the Care and Use of Laboratory Animals. All animal experiments in this study were reviewed and approved by the Institutional Animal Care and Use Committee of Kato Ladies Clinic (K004 and K007). All efforts were made to minimize the number of animals used and their suffering.

### 2.2 Embryo collection and culture

Female mice (C57BL/6J) aged 4–5 weeks (*n* = 105) were superovulated by intraperitoneal injection of 5 IU equine chorionic gonadotropin (eCG, ASKA Pharmaceutical Co., Ltd.), followed by 5 IU human chorionic gonadotropin (hCG, ASKA Pharmaceutical Co., Ltd.) 48 h later.^12^ On the night of the hCG injection, females were mated with C57BL/6J males to obtain fertilized embryos. The mice were sacrificed 44–48 h after hCG administration. A total of 1263 two‐cell stage embryos were collected from the oviducts of 105 mice. Two‐cell embryos were then cultured in KSOM + AA media at 37°C in 5% CO_2_ and 95% air. Twenty‐six embryos were cultured in a time‐lapse incubator to observe blastocoel re‐appearance and blastocyst re‐expansion after TE biopsy (Astec, Fukuoka). Embryonic images were collected every 15 min. The first frame when the blastocoel reappeared after the biopsy was annotated as “blastocoel re‐appearance.” The time when the blastocysts fully re‐expanded after the biopsy was annotated as “re‐expanded.” The blastocysts which exhibited blastocoel re‐appearance but not re‐expansion were categorized as “re‐expanding” (Figure S1).

### 2.3 Trophectoderm biopsy

All expanded blastocysts with herniating TE cells were used for the biopsy of herniating TE cells 3 days after the culture. Five to seven TE cells located apart from the inner cell mass were gently aspirated and separated from the blastocyst using a laser system (Cooper Surgical, Inc.) through a zona pellucida opening created by the laser at the four‐cell stage. The biopsied cells were stained with 1‐mg/ml Hoechst 33342 (Sigma Aldrich) and observed using a BZ‐X800 fluorescence microscope (Keyence). The blastocysts were vitrified at 0, 1, 2, 3, 4, 6, and 24 h after TE biopsy or non‐TE biopsy (Figure S2).

### 2.4 Vitrification and warming

Vitrification and warming were performed using Cryotop^®^ (Kitazato Corporation), as previously described.^13^ Briefly, blastocysts were equilibrated in an equilibrium solution consisting of 7.5% (v/v) ethylene glycol and 7.5% (v/v) dimethyl sulfoxide for 15 min. Blastocysts were then transferred to a vitrification solution consisting of 15% (v/v) ethylene glycol, 15% (v/v) dimethyl sulfoxide, and 0.5 M sucrose for 1.5 min. Then, they were placed on the Cryotop and immediately plunged into liquid nitrogen. For warming, the Cryotop was placed in a warming solution of 1.0 M sucrose at 37°C for 1 min. The blastocysts were then removed from the warming solution and transferred to a diluent solution of sucrose (0.5 M) at 26–28°C. After 3 min, the cells were transferred to a washing solution without sucrose. For final dilution, blastocysts were transferred to washing solution for 1 min. The survival rate at 24 h after warming was examined at control and biopsy groups at 0, 2, 4, 6, and 24 h after TE biopsy.

### 2.5 Outgrowth

The blastocyst outgrowth assay was performed as previously described, with slight modifications.^14^ The blastocysts after warming at control and biopsy groups at 0, 1, 2, 3, and 4 h after TE biopsy were placed on fibronectin‐coated dishes and cultured for 120 h at 37°C in 5% CO_2_ and 95% air for the outgrowth culture assay. When trophoblast cells grew outward from the blastocysts and the trophoblast cells became visible, these embryos were designated as adhesion‐initiating blastocysts. Blastocyst adhesion was evaluated by gentle pipetting, and the blastocyst outgrowth area was measured 120 h after commencing culture using NIS Elements D Imaging Software (Nikon).

### 2.6 Embryo transfer

The blastocysts in the control and biopsy groups at 0, 1, and 4 h after TE biopsy were transferred to the uteri of pseudo‐pregnant female Institute of Cancer Research (ICR) mice.^15^ The pseudo‐pregnancy mice were mated vasectomized ICR male mice to induce pseudo‐pregnancy (Day1 = vaginal plug). Nine to twelve blastocysts were transferred into the uteri at 3 days post‐coitum, and the pregnant females were sacrificed at 19 days post‐coitum.

### 2.7 Statistical analysis

Statistical analyses were performed using JMP software (SAS Inc.). The chi‐squared test was used to analyze the data in terms of survival rate, re‐expansion, and blastocyst adhesion rate. Fisher's exact probability test was used for comparisons when the expected values were <5. The blastocyst outgrowth area was compared using Student's *t*‐test or one‐way analysis of variance, and statistical significance was determined using Tukey's test for post hoc analysis. Statistical significance was set at *p* < 0.05.

## 3 RESULTS

### 3.1 Blastocyst viability after vitrification and thawing

The blastocyst survival rates after thawing were comparable between the control and biopsy groups at 0, 2, 4, 6, and 24 h after TE biopsy (Table 1). To evaluate the competence of blastocyst adhesion, an outgrowth assay was performed (Table 2 and Figure S3). No significant differences in blastocyst adhesion rates were observed among the experimental groups (Table 2). However, trophoblast outgrowth was significantly less extensive in the 1‐h biopsy group than in the control, 0‐h biopsy, and 4‐h biopsy groups (*p* = 0.0304, *p* = 0.0058, and *p* = 0.0029, respectively; Table 2). Although the area in the 2‐h biopsy group tended to be smaller than that in the 0‐h and 4‐h biopsy groups (*p* = 0.0883 and *p* = 0.0529, respectively; Table 2), the differences were not significant. Conversely, the area values in the 0‐h and 4‐h biopsy groups were comparable to those in the control group. The pup rate after the blastocyst transfer was significantly lower in the 1‐h group than those in the control, 0‐h biopsy, and 4‐h biopsy groups (*p* = 0.0006, *p* = 0.0081, and *p* = 0.0006, respectively; Table 3).

**TABLE 1:** Survival rate of vitrified‐warmed blastocysts after trophectoderm (TE) biopsy

Experimental group	Time interval to vitrification	No. of blastocysts examined	No. of blastocysts survived after warming (%)
Control	0‐h	55	54 (98.2)
	2‐h	59	59 (100)
	4‐h	55	55 (100)
	6‐h	34	34 (100)
	24‐h	55	52 (94.5)
Biopsy	0‐h	54	54 (100)
	2‐h	55	52 (94.5)
	4‐h	55	55 (100)
	6‐h	37	37 (100)
	24‐h	54	51 (94.4)

**TABLE 2:** Effects of different time intervals from trophectoderm (TE) biopsy to vitrification on blastocyst outgrowth

Experimental group	No. of blastocysts examined	No. of blastocysts adhesion on the dish (%)	Outgrowth are after 120 h of culture (×10^5^ μm^2^)
Control	23	23 (100)	5.7^a^
0‐h biopsy	27	27 (100)	6.0^a^
1‐h biopsy	27	26 (96.3)	4.4^b^
2‐h biopsy	27	27 (100)	5.0^ab^
3‐h biopsy	27	27 (100)	5.3^ab^
4‐h biopsy	27	27 (100)	6.1^a^

Note

Values within each column with different superscripts (a, b) are significantly different from each other (*p *< 0.05).

**TABLE 3:** Effect of different time intervals from trophectoderm (TE) biopsy to vitrification on blastocyst on in vivo developmental potential after blastocyst transfer

Experimental group	No. of recipient mice	No. of transferred blastocysts	No. of pups (%)
Control	13	137	64 (46.7)^a^
0‐h biopsy	12	118	50 (42.4)^a^
1‐h biopsy	12	119	31 (26.1)^b^
4‐h biopsy	13	132	62 (47.0)^a^

Note

Values within each column with different superscripts (a, b) are significantly different from each other (*p *< 0.05).

### 3.2 Fine analysis of blastocyst morphokinetics after TE biopsy

Twenty‐six blastocysts were monitored in a time‐lapse incubator after TE biopsy. The blastocoel was observed in 34.6% of blastocysts (9/26) at 0.25 h after TE biopsy. The rate of blastocoel re‐appearance was significantly increased at 1 h after TE biopsy, when compared to that observed at 0.25 h (*p* = 0.0054), and all blastocysts exhibited the blastocoel at 5 h after TE biopsy (Table 4). The average time required for the re‐appearance of the blastocoel was 1.1 ± 0.2 h. The re‐expanded blastocyst was first observed at 1 h after TE biopsy, and the re‐expansion rate was significantly increased at 3 and 4 h after TE biopsy (*p* = 0.0385 and *p* = 0.0479, respectively; Table 4). The average time required for re‐expansion was 3.4 ± 0.3 h, and re‐expanding blastocysts were observed at a high incidence 1–2 h after TE biopsy (Table 4).

**TABLE 4:** Morphological alteration of blastocysts after trophectoderm (TE) biopsy

	0 h	0.25 h	0.5 h	0.75 h	1 h	2 h	3 h	4 h	5 h	>6 h
No. of blastocoel re‐appearance	0	9^a^	14^ab^	16^bc^	19^bc^	23^cd^	24^cd^	25^d^	26^d^	26^d^
No. of re‐expanding blastocysts	0	9^ab^	14^bc^	16^bc^	18^c^	18^c^	12^abc^	6^ad^	3^d^	0
No. of re‐expanded blastocysts	0	0	0	0	1^a^	5^a^	12^b^	19^c^	23^cd^	26^d^

Note

Values within each column with different superscripts (a–d) are significantly different from each other (*p *< 0.05).

## 4 DISCUSSION

In this study, we examined the correlations of different time intervals from TE biopsy to vitrification with the blastocyst survival rate and blastocyst outgrowth ability. Our findings indicated that the time interval from TE biopsy to vitrification did not correlate with the survival rate after warming. Although the rate of blastocyst adhesion to fibronectin‐coated dishes was not affected by the time interval between TE biopsy and vitrification, the area of trophoblast outgrowth was significantly decreased in the 1‐h biopsy when compared to that in the control, 0‐h biopsy groups. Furthermore, the number of re‐expanding blastocysts increased 1–2 h after the TE biopsy.

We first examined whether the time interval between TE biopsy and vitrification affects blastocyst viability after warming. No significant difference in the survival rate after warming was observed regardless of the intervention of TE biopsy or the time interval from TE biopsy to vitrification, which is in accordance with the previous finding that blastocyst survival after warming is not affected by the biopsy procedure.^7^ Moreover, we assessed TE viability using an outgrowth model. The outgrowth area in the 0‐h biopsy group was comparable to that in the control (non‐biopsied) group. In contrast, the area in the 1‐h biopsy group was significantly smaller than that in the control and 0‐h biopsy groups. Furthermore, when the blastocysts were cultured for more than 3 h after TE biopsy, the outgrowth area recovered to a level comparable to that of the control and 0‐h biopsy groups. These results suggest that blastocyst vitrification should be performed within 1 h or more than 3 h after TE biopsy.^1^, ^9^, ^10^ Secondly, we conducted blastocysts transfer into mice. Similar to the results of the outgrowth area, the number of pups obtained of 0‐h biopsy and 4‐h biopsy groups was comparable to that of the control group, and the 1‐h biopsy group was significantly lower. Furthermore, these results suggest that time interval from TE biopsy to vitrification affects developmental competence.

To determine why vitrification at 1–2 h after TE biopsy adversely affects blastocyst outgrowth, we observed the morphokinetics of biopsied blastocysts using time‐lapse systems. The times required for the re‐appearance of blastocoel and complete re‐expansion after TE biopsy were 1.1 ± 0.2 h and 3.4 ± 0.3 h respectively. During TE biopsy, TE cells were separated from blastocysts using laser systems, indicating that a part of the TE may be affected by laser‐related thermal damage. Therefore, repair of components at the cellular level, formation of tight junctions between cells, and initiation of re‐expansion may be required for TE cells at the biopsy site following the procedure. Although we did not investigate the repair of cellular components or tight junction formation in the present study, we speculate that these processes were not associated with the impairment of outgrowth at 1 h after biopsy, as the re‐expansion of blastocysts had already been initiated by the 1‐h mark. Contrastingly, the status of blastocyst re‐expansion may be associated with impaired outgrowth. Approximately 70% of biopsied blastocysts were in the process of re‐expansion 1–2 h after the biopsy. This indicates that vitrification in the process of re‐expansion may lead to impairment in the competence of trophoblast migration after warming. The molecular mechanism that vitrification of re‐expanding blastocysts has adverse effects is unclear. However, we hypothesized that one of the reasons would be the alteration of intra‐cellular osmolality during the vitrification. The expansion of blastocoel is regulated by the membrane channels, for example, the Na+/K+‐ATPase enzyme regulates the water inflow into the cytoplasm by altering the intra‐cellular osmolality.^16^ Therefore, if the blastocysts were vitrified in re‐expansion, the intra‐cellular osmolality may be adversely affected by the vitrification procedure, leading to the disturbance of intra‐cellular osmolality regulation and re‐expansion after warming. Additionally, the intracytoplasmic ATP is consumed for the blastocyst re‐expansion since Na+/K+‐ATPase enzyme needs to work^17^; therefore, we considered that the vitrification should be performed prior to the activation of Na+/K+‐ATPase enzyme. Further studies are warranted to reveal the reason why vitrification during embryo expansion adversely affects the subsequent development of blastocysts. Furthermore, cryopreservation of large embryos is challenging because the embryonic surface area per volume is low, resulting in insufficient exchange of water and cryoprotectants.^18^, ^19^ Moreover, previous studies have reported that artificial shrinkage of the blastocoel before the vitrification procedure improves embryo viability and developmental potential.^20^, ^21^ Taken together, these findings suggest that vitrification of biopsied blastocysts should be performed with minimal embryonic volume immediately after TE biopsy and before initiating re‐expansion.

This study had certain limitations. First, we investigated the effects of different time intervals from TE biopsy to vitrification on blastocyst viability using a mouse model. Since the results demonstrated in this study may vary when human embryos are used, further investigations using human embryos are required to validate our findings. However, the mechanism of outgrowth to fibronectin, such as the interaction of integrins and fibronectin, is similar between mice and humans^22^, ^23^, ^24^, ^25^; thus, we consider that similar phenomena may occur in human embryos. Second, we have not examined the correlation between the number of biopsy trophoblasts and the outgrowth area. Furthermore, we have not counted the number of TE cells in the blastocysts before biopsy to avoid the exposure of ultraviolet light to the embryos. The authors considered that the number of TE cells in the blastocysts post‐biopsy is more associated with the blastocyst outgrowth than the number of TE cells biopsied; therefore, further studies that assess the correlation between the blastocyst outgrowth and the number of TE cells post‐biopsy are required.

In conclusion, the present findings indicate that the time interval from TE biopsy to vitrification of blastocysts does not affect embryo survival, but that vitrification during re‐expansion impairs blastocyst outgrowth. In the clinical setting, vitrification of biopsied blastocysts during re‐expansion may decrease the pregnancy rate by decreasing the competence of post‐implantation development. To prevent this occurrence, blastocyst vitrification should be performed immediately after TE biopsy.

## CONFLICT OF INTEREST

The authors have no conflicts of interest to declare.

## ETHICAL APPROVAL

All animal experiments in this study were reviewed and approved by the Institutional Animal Care and Use Committee of Kato Ladies Clinic (K004 and K007).

## HUMAN RIGHTS STATEMENTS AND INFORMED CONSENT

This article does not contain any studies with human subjects performed by any of the authors.

## ANIMAL STUDIES

All mouse experiments were conducted in accordance with the guidelines of the Institutional Animal Care and Use Committee of Kato Ladies Clinic, which are based on the Animal Welfare Act Regulations and the Guide for the Care and Use of Laboratory Animals. All efforts were made to minimize the number of animals used and their suffering.

## Supporting information

Figure S1

Figure S2

Figure S3
