# Supplementary figures and images for: Time from trophectoderm biopsy to vitrification affects the developmental competence of biopsied blastocysts
Source: Reprod Med Biol. 2022 Jan 29;21(1):e12439. doi: 10.1002/rmb2.12439 (PMC8967302; doi:10.1002/rmb2.12439)

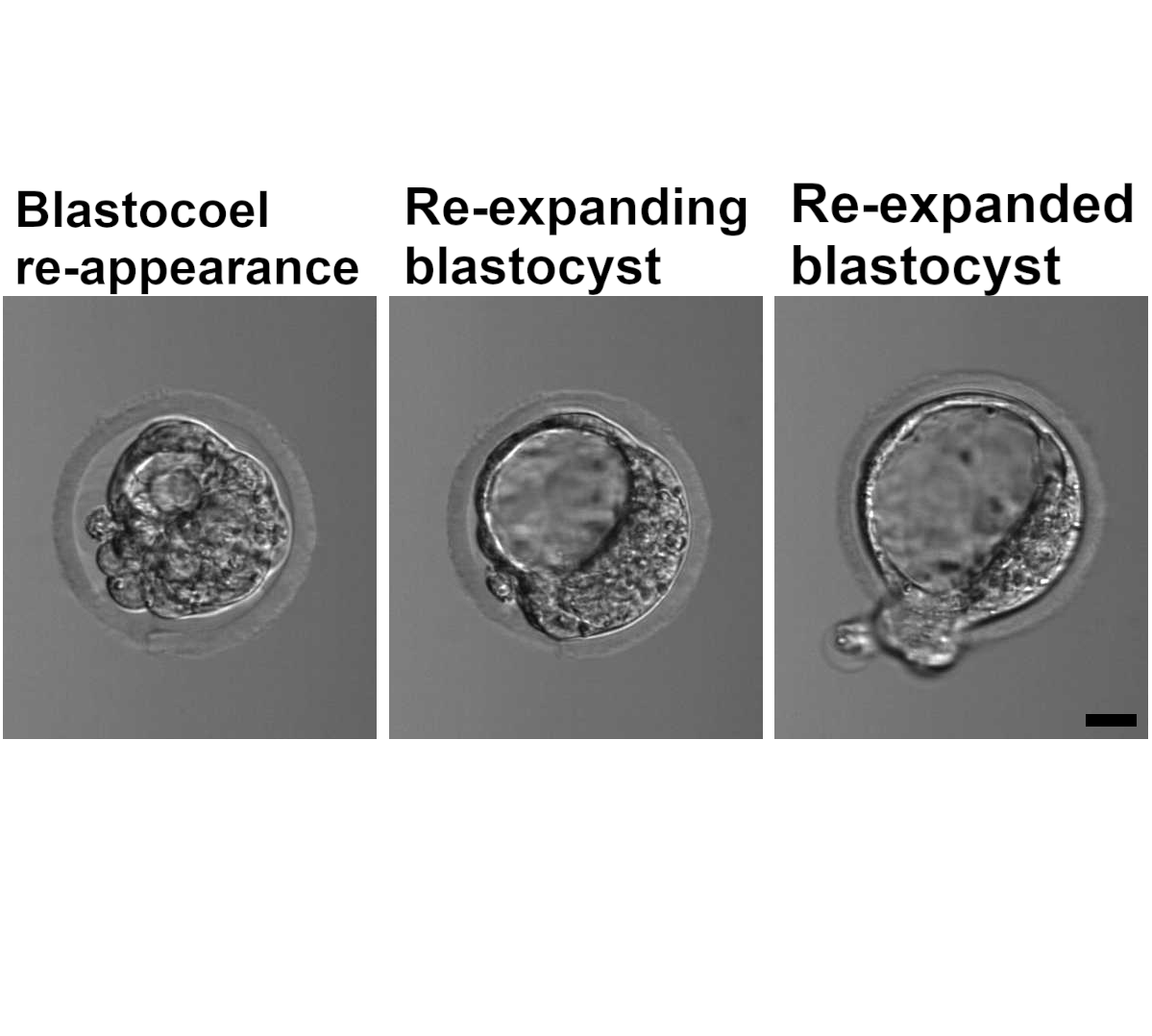

Supplement: Supplementary file 1 — Figure S1 [file RMB2-21-e12439-s001.tif]

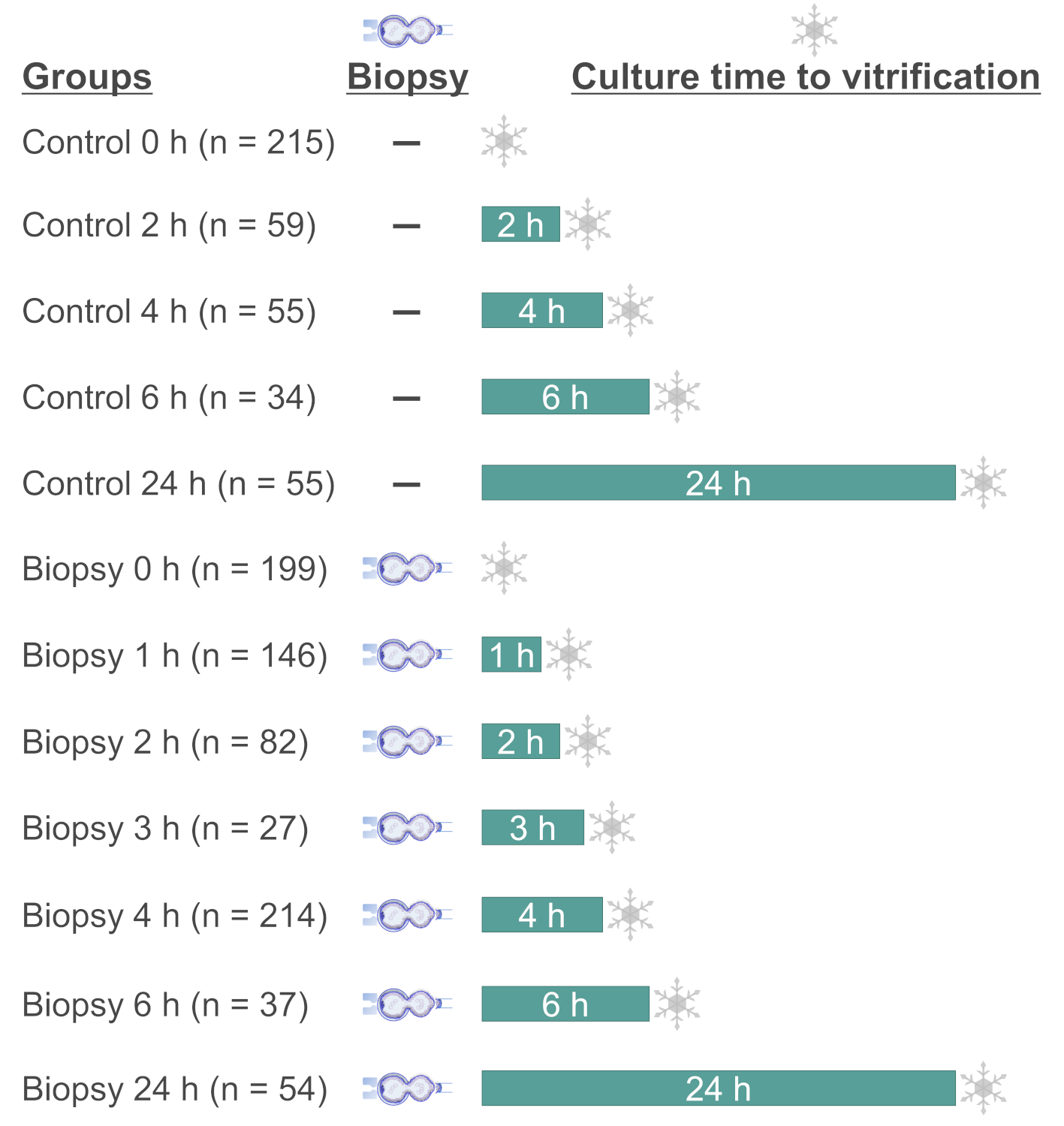

Supplement: Supplementary file 2 — Figure S2 [file RMB2-21-e12439-s002.tif]

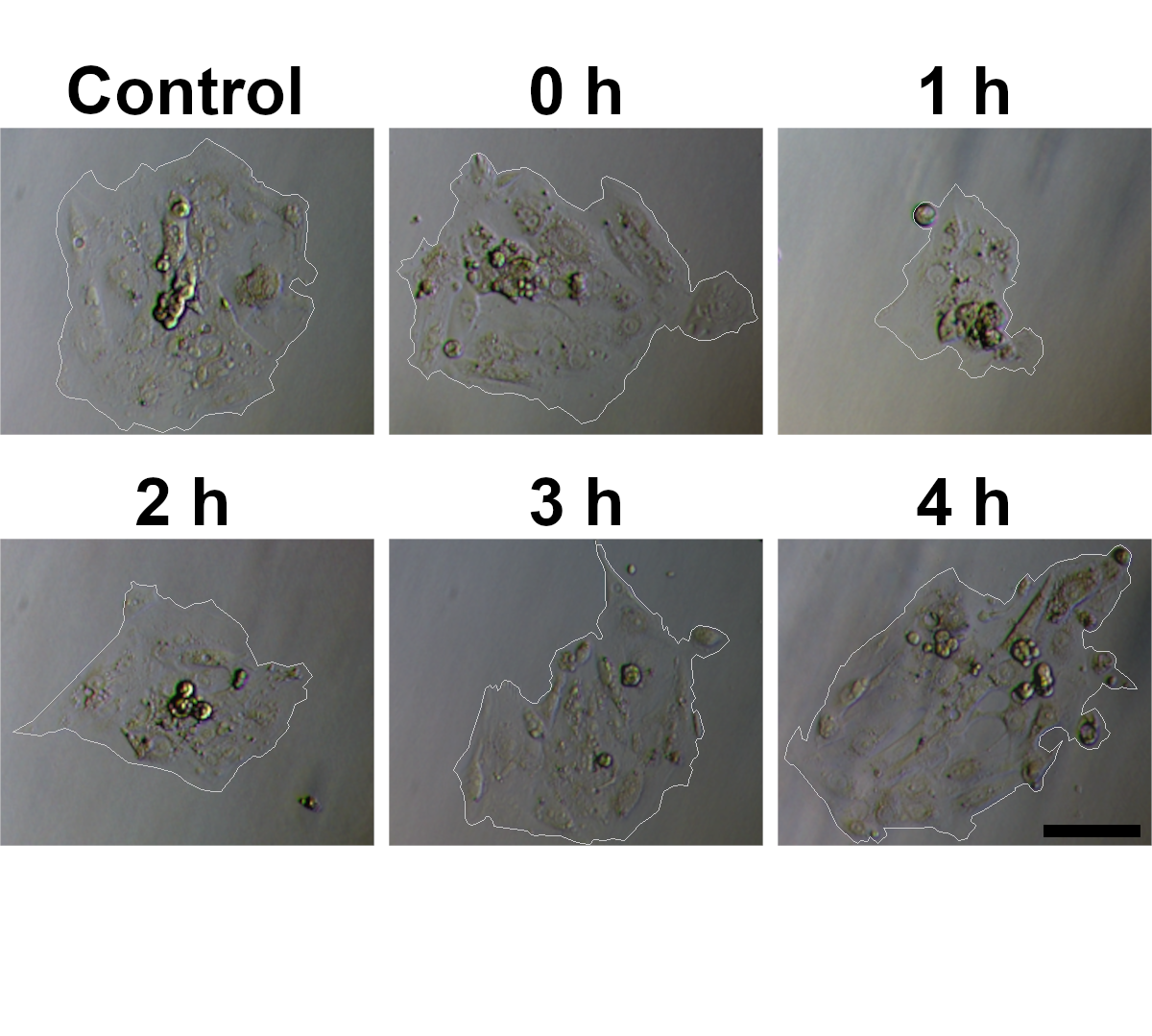

Supplement: Supplementary file 3 — Figure S3 [file RMB2-21-e12439-s003.tif]
